# Fulminant arterial vasculitis as an unusual complication of disseminated staphylococcal disease due to the emerging CC1 methicillin-susceptible *Staphylococcus aureus* clone: a case report

**DOI:** 10.1186/s12879-019-3933-3

**Published:** 2019-04-03

**Authors:** Charles Vidal, Florence Moulin, Xavier Nassif, Louise Galmiche, Delphine Borgel, Alain Charbit, Capucine Picard, Jean-Paul Mira, Olivier Lortholary, Anne Jamet, Julie Toubiana

**Affiliations:** 1 Department of Microbiology, Necker Enfants-malades hospital, APHP, Paris Descartes University, Paris, EU France; 2 Department of Pediatric Intensive Care Unit, Necker Enfants-Malades Hospital, APHP, Paris Descartes University, Paris, EU France; 3 Pathology Department, Necker Enfants-Malades Hospital, APHP, Paris Descartes University, Paris, EU France; 4 Department of Hematology, Necker Enfants-Malades Hospital, APHP, Paris Descartes University, Paris, EU France; 5 Necker-Enfants-Malades Institute, INSERM U1151; CNRS UMR8253, Paris, France; 6 Center for the Study of Primary Immunodeficiencies, Necker Enfants Malades Hospital, APHP, Paris Descartes University, Paris, EU France; 7 IHU Imagine, Laboratory of Human Genetics of Infectious Diseases, INSERM U1163, Paris, EU France; 8 Medical Intensive Care Unit, Cochin Hospital, AP-HP, Paris Descartes University, Paris, EU France; 9 Department of Infection, Immunity and Inflammation, Institut Cochin, INSERM U1016, Paris, EU France; 10 Department of Infectious Diseases and Tropical Medicine, Necker Enfants-Malades Hospital, Necker-Pasteur Infectious Diseases Center, Université Paris Descartes, IHU Imagine, Paris, EU France; 11 Department of General Pediatrics and Pediatric Infectious Diseases, Necker Enfants-Malades Hospital, APHP, Paris Descartes University, 149 rue de Sèvres, 75015 Paris, EU France

**Keywords:** *S. aureus*, Sepsis, Vasculitis, Virulence, Genetic susceptibility, Polymorphism, Case report

## Abstract

**Background:**

*Staphylococcus aureus* has emerged as a leading cause of invasive severe diseases with a high rate of morbidity and mortality worldwide. The wide spectrum of clinical manifestations and outcome observed in staphylococcal illness may be a consequence of both microbial factors and variability of the host immune response.

**Case presentation:**

A 14-years old child developed limb ischemia with gangrene following *S. aureus* bloodstream infection. Histopathology revealed medium-sized arterial vasculitis. The causing strain belonged to the emerging clone CC1-MSSA and numerous pathogenesis-related genes were identified. Patient’s genotyping revealed functional variants associated with severe infections. A combination of virulence and host factors might explain this unique severe form of staphylococcal disease.

**Conclusion:**

A combination of virulence and genetic host factors might explain this unique severe form of staphylococcal disease.

## Background

*Staphylococcus aureus* is a major human pathogen and a global healthcare issue. Humans are a natural reservoir of *S. aureus*, which can occasionally cause diseases that range in severity from minor skin infections to severe cases of pneumonia, bacteremia and septic shock [1]. The severity and outcome of the infection relies on bacterial virulence, as *S. aureus* is known to have a wide arsenal of components that contribute to the pathogenesis of infection. *S. aureus* is the cause of septic shock through cell wall components eliciting production of inflammatory cytokines through TLR2 pathway activation in innate immune cells [2]. *S. aureus* also contains several toxins that are able to potentiate host-inflammatory response, target and injure leukocytes and tissues, and inhibit bacterial clearance [3]. Finally, *S. aureus* avidly adheres to endothelial cells and platelet-fibrin thrombi involved in the physiopathology of endocarditis or thrombophlebitis [4]. Recently, the role of genetic factors of the host has been extensively studied, revealing their influence on the susceptibility to or the severity of sepsis. Rare monogenic inborn errors of immunity were found to predispose to severe infectious diseases, such as mutations that impair NF-κB responses [5]. In parallel, common variants of genes involved in innate immune response, inflammation and coagulation during sepsis were found to be associated with severity of bacterial infections [6, 7]. We report here a severe and atypical phenotype of infection due to *S. aureus.* The young patient developed medium-sized arterial infectious vasculitis that led to ischemia and gangrene. We intended to decipher pathogen and host factors that could explain this dramatic presentation.

## Case presentation

### Clinical case

The patient was a 14-year-old male child without notable past medical history and no recent travel. On admission, he had fever, lethargy and diarrhea, and physical examination revealed fever (39 °C), tachycardia, blood pressure 65/35 mmHg and poor peripheral perfusion. Several boils were observed on the right elbow. He had subnormal white blood cell count 2.8 × 10^9^ / L with low lymphocyte count (13%), a moderate thrombocytopenia and raised CRP (347 mg/L) and procalcitonin plasma levels (279 ng/mL). The diagnosis of septic shock with a possible associated toxic mechanism was retained and intravenous (i.v.) cefotaxime and clindamycin were started with concomitant volume expansion. In the Intensive Care Unit, the patient was intubated as he became confused, needed norepinephrine, inotropic support by epinephrine, and required hemofiltration. Blood cultures showed methicillin susceptible *S. aureus* (MSSA) and treatment was switched to i.v. cloxacillin, clindamycin, and gentamicin. Complementary explorations revealed multiple septic pulmonary abscesses, an abscess of the left occipital lobe, and the absence of endocarditis. In view of septic emboli, clindamycin was switched to fosfomycin in order to have a better cerebral diffusion. The patient then developed ischemia of the four limbs without any purpuric lesion or signs of disseminated intravascular coagulation, which unfortunately rapidly progressed to dry gangrene of the left toes and of the right leg requiring amputation at day 11 after admission. The clinical status of the patient improved slowly. Catecholamines were stopped at day 6, the patient got extubated at day 11, and he recovered an efficient renal function 5 weeks after his admission. He had no serious neurological injury and a control cerebral MRI was normal three months later.

### Histopathology, biological and genetic investigations

**Fig. 1:**
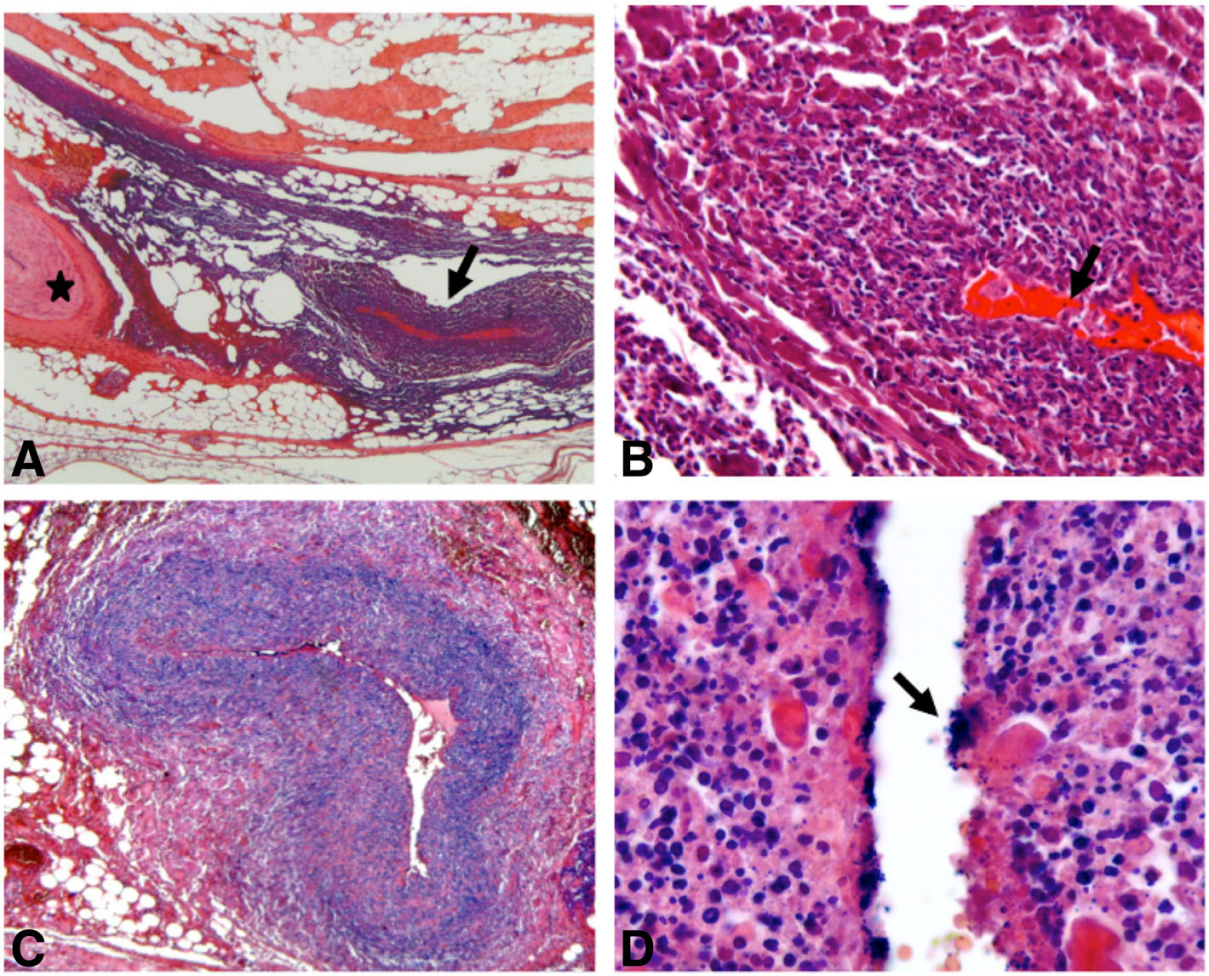
Histopathology of the amputation specimen. **a** Coexistence of normal (star) and pathologic medium-sized arteries (arrow) in necrotic and ischemic fibro-adipose tissue. Focal infiltration by inflammatory cells is noted. **b** and **c** Pathologic artery occluded by fibrinous thrombus (arrow). Arterial wall is replaced by cellular debris without residual endothelium. Surrounding tissues are necrotic and ischemic. **d** Gram staining shows bacterial colonization of arterial wall. Numerous bacteria (arrow) are found around the lumen

Our initial hypothesis underlying this atypical phenotype was the association of a vasospasm due to high doses of vasopressors and microcirculation disorders due to sepsis. We therefore analyzed the amputated specimen by light microscopy. Samples of left and right amputations were formalin fixed, paraffin embedded, and cut into 3 μm-thick sections. Sections were stained with Hematoxylin and Eosin, Gram, Periodic Acid Schiff and Grocott staining. The histopathologic analysis revealed perivascular infiltrates of mononuclear cells and neutrophils with arterial involvement (medium-sized vessels) without any capillary lesion (Fig. 1a and b). Furthermore, the arterial endothelium was destroyed and the walls were invaded by Gram-positive cocci with subsequent thrombosis (Fig. 1c and d).

In order to decipher mechanisms underlying these clinical and histopathological findings, pathogen virulence and host factors were analyzed. A whole-genome shotgun library was prepared with Nextera XT Kit (Illumina, San Diego, CA, USA) and sequenced on the MiSeq Illumina sequencing platform (2 × 150 bp paired-end reads). All reads were screened by mapping to known alleles of virulence genes using the Short Read Sequence Typing for Bacterial Pathogens program (SRST2) [8]. Virulence gene allele sequences were retrieved from the virulence factor database (VFDB, http://www.mgc.ac.cn/VFs/). SRST2 was also used to define the sequence type of the strain. Antibiotic-resistance predictions were performed directly from raw reads using “Mykrobe predictor *S. aureus*” tool [9]. All generated sequences are available at NCBI’s BioProject database under accession number PRJNA315766 (http://www.ncbi.nlm.nih.gov/bioproject/PRJNA315766). The sequenced *S. aureus* isolate was a methicillin-susceptible strain harboring a staphylococcal cassette chromosome (SCC). The patient’s strain genome encoded numerous pathogenesis-related genes. The virulence gene equipment of the strain notably included several cytotoxin genes encoding the gamma-hemolysin and the Panton-Valentine (LukSF-PV), LukED and LukGH leukotoxins. It also included genes involved in adhesion and biofilm formation, genes encoding various enterotoxins (A, H, K, and Q) and superantigens, exoproteases and immune response evasion proteins (Table 1). The patient’s strain belonged to the clonal complex CC1-MSSA-SCC*fus* [PVL+].

**Table 1:** Significant virulence genes identified by whole-genome sequencing of the clinical isolate

Gene name	Description
Adhesion factors	
*cna*	Collagen-binding adhesin precursor
*ebpS*	Elastin-binding protein
*clfA, clfB*	Clumping factors
*fnbA, fnbB*	Fibronectin-binding proteins
*sasG*	*Staphylococcus aureus* surface protein G
*sdrC, sdrD, sdrE*	Serine–aspartate repeat proteins
Biofilm-associated genes	
*icaADB*	intercellular adhesion proteins
*icaR*	*ica* operon transcriptional regulator
Cytotoxins	
*hlgABC*	gamma-hemolysin
*lukED*	leukotoxin ED
*lukGH*	leukotoxin GH
*lukSF-PV*	Panton-Valentine leukocidin
Enterotoxins	
*sea, seh*	enterotoxins
*sek, seq*	enterotoxin-like proteins
Staphylococcal superantigen-like	
*set*(s)	superantigen-like proteins
Proteases	
*splABCDEF*	serine-like proteases
*sspA*	Serine V8 protease
*sspB*	Staphopain B cysteine protease
Immune response evasion	
*efb/fib*	extracellular fibrinogen binding protein precursor
*scn*	staphylococcal complement inhibitor (SCIN)
*sak*	staphylokinase precursor

Then, thrombophilic factors and immunity of the patient were examined. Initial low circulating protein antithrombin, C and S returned to normal after one month, and other factors associated with hereditary thrombophilia including activated protein C resistance, lupus anticoagulant and prothrombin G20210A mutation, were absent. Lymphocyte subsets of the patient were determined by routine flow cytometry, serum levels of the IgM, IgA, IgG, and IgG subclasses were assessed by standard nephelometry techniques, and complement was measured via enzyme-linked immunosorbent assay (ELISA). Activation of complement pathways was normal as well as B, T and NK lymphocyte cell count and immunoglobulin levels. Then, activation of cells in whole-blood of the patients were determined after granulocyte isolation by Ficoll density gradient centrifugation. Cells were activated with Toll like receptors agonists, stained with anti-CD62L-FITC, and analyzed by flow cytometry. In vitro stimulation of the patient’s peripheral blood mononuclear cells by LPS showed a standard production of cytokines IL-6 and IL-10 by ELISA.

Finally, functional common polymorphisms associated with severe infections and septic shock (PAI-1, rs1799768; MIF, rs755622; ACE rs17326674 IL6, rs1800795; TNF rs1800750, rs1800629, rs361525, and rs909253; TLR2, rs5743708 and rs5743704, TLR4, rs4986790; TLR5, rs5744168; IRAK1, rs1059703; IKB, rs2233406 and rs3138053; FCgammaRIIA, rs1801274 and CFH, rs1065489) [6, 7] were genotyped using TaqMan Single Nucleotide Polymorphism Genotyping Assays as previously described [7]. Genomic DNA was extracted from mononuclear cells using MagnaPure Compact automate (Roche Diagnostics®). The SNP genotyping revealed that the patient was homozygous for four potential deleterious variants of *IRAK1*, *MIF*, *ACE*, and *PAI-I* (Table 2).

**Table 2:** Genotype findings of the patient at all loci tested. Homozygous potential deleterious variants were observed for four genes: *IRAK1, MIF, ACE* involved in inflammation and *PAI-1* in thrombotic events

Gene name	Ref	WT/WT	WT/M	M/M
*PAI-1*	rs1799768			x
*MIF*	rs755622			x
*ACE*	rs17326674			x
*IL-6*	rs1800795	x		
*TNF*	rs1800750	x		
	rs1800629	x		
	rs361525	x		
	rs909253	x		
*TLR2*	rs5743708	x		
*TLR2*	rs5743704	x		
*TLR4*	rs4986790	x		
*TLR5*	rs5744168	x		
*IRAK1*	rs1059703			x
*IKB*	rs2233406	x		
	rs3138053	x		
*FcgammaRIIA*	rs1801274	x		
*CFH*	rs1065489	x		

*PAI-1* Plasminogen activator inhibitor 1, *MIF* Macrophage migration inhibitor factor, *ACE* Angiotensin converting enzyme, *IL-6* Interleukin 6, *TNF* Tumor Necrosis Factor, *TLR* Toll like receptor, *IRAK1* Interleukin-1 receptor associated kinase, *IΚB* Inhibitory protein of nuclear factor-κB, *FCgammaRIIA* Fc receptor for IgG, *CFH* Complement factor H, *WT* Wild type concerns the frequent allele, *M* Mutation concerns the variant rare allele, *Ref* Reference number for the studied SNP

## Discussion and conclusions

To our knowledge, it is the first reported case of vasculitis occurring during *S. aureus* disseminated infection affecting the medium-sized arteries without any capillary injury. Few cases of extremity necrosis induced by *S. aureus-*producing superantigens have been reported in both adults and children but necrosis was associated with purpura fulminans caused by lesions of the capillaries and subsequent thrombosis [10, 11]. Most of these patients had a fatal outcome due to septic shock or displayed gangrene of the distal extremities. This uncommon phenotype could be the consequence of the interaction of a virulent pathogen and a susceptible host.

Recently, an emergence of CC1-MSSA has been reported [12, 13]. However, to our knowledge, no severe cases due to CC1-MSSA clone had been reported so far. The PVL toxin and the four other enterotoxins isolated from our patient are likely to be responsible for a massive cytokine release with subsequent toxic shock syndrome and transient alteration of the immune system [14]. Staphylococcal superantigens could also be responsible for an inflammatory vessel vasculitis. However, the association between superantigen production and poor prognosis of MSSA infection is still under debate [15]. *S. aureus* is also known to interact with the endovascular system through the expression of numerous adhesins leading to specific infection such as infective endocarditis, suppurative thrombophlebitis, or vascular graft infection [4]. *ClfB*, *cna* and *sea* genes that encode respectively clumping factor B, collagen adhesin and enterotoxin A, were present in the patient strain. However, the venular endothelium seems to be the predominant target for *S. aureus* binding. We only provided here a list of virulence genes present in the strain, but variations in the expression of these genes or in protein production could be responsible for the dramatic clinical presentation and the extreme prothrombotic phenotype observed in our patient [16].

Associated with virulent factors, our data argue for direct role of unusual immune and coagulation system responses of the host. The expression of adhesion molecules on endothelial cell wall is up-regulated during sepsis and might help colonization by the pathogen [4]. A hyperinflammatory state associated with dysfunction of the endothelium in sepsis can lead to the disturbance of the coagulation balance and subsequent thrombosis [2, 17]. This may be facilitated by the presence of functional variants of genes involved in inflammatory response [6]. Indeed, our patient was homozygous for 4 polymorphisms associated with higher susceptibility and/or severity of severe sepsis: *IRAK1*, *ACE, PAI-1* and *MIF* genes [6, 7, 18, 19]. In particular, *PAI-1* (gene encoding for plasminogen activator inhibitor-1) variant is associated with decreased fibrinolysis and a higher risk of amputation of the extremities in septic shock [6, 18]. Even if the final effect of combination of these four potentially deleterious polymorphisms is unknown, it might explain in part the increased inflammatory and pro-coagulant state, and the severity of the staphylococcal infection in our patient.

In conclusion, disseminated *S. aureus* infection, apart from septic shock, could lead to arterial vasculitis and arterial thrombosis, with severe consequences such as limb amputation. The combination of pathogen virulence and genetic variability of the host response probably explain the dramatic severity of this infection. The investigation of predisposing factors might help for future tailor-made adjunctive therapy in sepsis.

## Abbreviations

ACE: Angiotensin I-converting enzyme
CRP: C-reactive protein
ELISA: Enzyme-liked immunosorbent assay
IRAK: Interleukin-1 receptor-associated kinase 1
MIF: Macrophage inhibition inhibitory factor
MRI: Magnetic resonance imaging
PAI-I: Plasminogen activator inhibitor type I
PVL: Panton-Valentine leukocidin
SCC: Staphylococcal cassette chromosome
SNP: Single polymorphism nucleotid
